# Preoperative myocardial fibrosis is associated with worse survival after alcohol septal ablation in patients with hypertrophic obstructive cardiomyopathy: A delayed enhanced cardiac magnetic resonance study

**DOI:** 10.3389/fcvm.2022.924804

**Published:** 2022-08-11

**Authors:** Youzhou Chen, Xingshan Zhao, Jiansong Yuan, Yan Zhang, Wei Liu, Shubin Qiao

**Affiliations:** ^1^Department of Cardiology, Beijing Jishuitan Hospital, Beijing, China; ^2^Department of Cardiology, Fuwai Hospital, National Center for Cardiovascular Diseases, Chinese Academy of Medical Sciences and Peking Union Medical College, Beijing, China; ^3^Department of Magnetic Resonance Imaging, Fuwai Hospital, National Center for Cardiovascular Diseases, Chinese Academy of Medical Sciences and Peking Union Medical College, Beijing, China

**Keywords:** hypertrophic cardiomyopathy, alcohol septal ablation, myocardial fibrosis, outcome, late gadolinium enhancement (LGE) MRI

## Abstract

**Background:**

Prior studies have shown that myocardial fibrosis can be detected by late gadolinium enhancement (LGE) of cardiac magnetic resonance (CMR) and might be associated with higher mortality risk in hypertrophic cardiomyopathy (HCM). The objective of this study was to examine the prognostic utility of CMR in patients with hypertrophic obstructive cardiomyopathy (HOCM) undergoing alcohol septal ablation (ASA).

**Materials and methods:**

We conducted a retrospective study which consisted of 183 consecutive patients with symptomatic drug-refractory HOCM who underwent CMR for assessment of myocardial fibrosis before ASA. The cardiovascular disease related survival was evaluated according to LGE-CMR status.

**Results:**

The cohort comprised 74 (40.4%) women with a mean age of 51 ± 8 years. Preoperative myocardial fibrosis was detected in 148 (80.9%) patients. After a median of 6 years (range 2–11 years) follow-up, adverse clinical events occurred in 14 (7.7%) patients. Multivariate-adjusted Cox regression analyses revealed that age [hazard ratio (HR) 1.142 (1.059–1.230), *p* = 0.001] and LGE [HR 1.170 (1.074–1.275), *p* < 0.001] were independent predictors of cardiovascular mortality during follow-up.

**Conclusion:**

Preoperative myocardial fibrosis measured by LGE-CMR was an independent predictor of increased adverse clinical outcomes in patients with HOCM undergoing ASA and could be used for the pre-operative evaluation of risk stratification and long-term prognosis after ASA in these patients.

## Introduction

Hypertrophic cardiomyopathy (HCM) is the common genetic inherited heart disease with a prevalence of about 0.2% (1). Dynamic left ventricular outflow tract (LVOT) obstruction is an important pathophysiologic phenomenon, with significant impact on symptoms such as dyspnea, angina, and prognosis of the obstructive HCM (HOCM). For those with drug-refractory symptoms, alcohol septal ablation (ASA) was introduced as an alternative therapeutic option to relieve LVOT obstruction, which is associated with a lasting clinical efficacy and long-term follow-up results (2). However, there are still a subgroup of patients with unsatisfactory responses after ASA such as having symptoms and residual LVOT obstruction (3, 4).

The areas of myocardial fibrosis are thought to constitute the substrate for life-threatening arrhythmia and adverse cardiac remodeling in HCM (5). Myocardial fibrosis as measured by late gadolinium enhancement (LGE) of cardiac magnetic resonance (CMR) is directly proportional to the extent of myocardial fibrosis which has been proved by histopathological studies (6). LGE is also related to HCM related adverse events, including progressive heart failure and sudden death (7). Little is known with respect to the impact of myocardial fibrosis before ASA on the clinical outcome after ASA. Additionally, whether ASA is also effective in patients with extensive septal scarring on CMR remains unknown (8).

The purpose of our study was to examine the impact of LGE on the clinical outcomes after ASA at long-term follow-up. We also assessed whether the amount of LGE can serve as a promising tool for the risk stratification in patients with HOCM undergoing ASA.

## Materials and methods

### Study population

This was an observational retrospective study approved by the institutional review committee of Fuwai Hospital, China. Between September 2005 and December 2014, 183 secutive patients with HCM were recruited for ASA to our center. The diagnosis of HCM were based on typical clinical and echocardiographic characteristics with unexplained ventricular myocardial hypertrophy occurring in the absence of any other accountable cardiac or systemic disease (2, 9). The indication for ASA was HOCM with New York Heart Association (NYHA) class III/IV even the optimal drug therapy, recurrent exercise-induced pre-syncope or syncope and a resting LVOT gradient of >50 mm Hg or >100 mm Hg during provocation (9, 10). All patients wrote the informed consent.

### Alcohol septal ablation procedure

The ASA technique has been previously described and was performed by the interventional cardiologists (11). Before the procedure began, a temporary pacemaker was placed in all patients. With the help of the contrast echocardiography, the septal arterial branch supplying the target septal area was identified. 1–3 mL of ethanol was injected into the artery supplying the culprit septal segments and the balloon was removed 5 min after the last alcohol injection (12). A successful procedure was defined as a reduction in the LVOT pressure gradient ≥ 50% of baseline (13). If the operator was not satisfied with the result, the whole procedure could be repeated in another septal branch.

### Cardiac magnetic resonance examination

All patients underwent the CMR examinations before the ASA procedure after enrolled in the Fuwai Hospital. The CMR examinations were performed on a 1.5T MRI machine (Siemens Medical Solutions, Erlangen, Germany) with a steady-state, free-precession breath-hold cines in 3 long-axis planes and sequential 10 mm short-axis slices (14, 15). LGE images were acquired 10–20 min after administration of 0.2 mL/Kg of gadolinium-DTPA (Magnevist, Schering; Berlin, Germany) using inversion-recovery sequences using a segmented phase-sensitive inversion recovery (PSIR) spoiled gradient echo sequence (16, 17). LGE-CMR imaging was acquired in short-axis views covering the LV from the mitral annular plane to the apex with 6 mm slice thickness and 1.6 mm gaps. Typical imaging parameters were: repetition time (TR) = 8 ms, echo time (TE) = 3.6 ms. Field of view (FOV) = 380 × 320 mm^2^, matrix = 256 × 162, temporal resolution = 40 ms. The inversion time was adjusted to optimally null signal from normal myocardium typically between 250 and 350 ms (17).

All CMR images were analyzed by two experienced radiologic technicians with commercially available software (Medis Medical Imaging Systems, Leiden, Netherlands). LV volume and mass measurements were acquired when the endocardial and epicardial contours were manually drawn in cardiac end-systole and diastole phase. LV mass was calculated by multiplying the volume of the myocardium calculated at end-diastole by the specific gravity of the myocardium (1.05 g/ml) (18). LV volume and mass measurements were indexed to body surface area. To measure the amount of LGE, all short-axis LV slices from base to apex were inspected to ensure an area of completely nulled myocardium. The mean signal intensity (and SD) of normal myocardium was calculated, and a threshold ≥ 6 SDs exceeding the mean was used to define areas of LGE (19, 20). To define LGE areas 6 SD threshold was used and the extent of LGE was expressed as of proportion of total LV mass (%LGE) (17).

### Study endpoints and definitions

The primary end points were defined as cardiovascular death, which included sudden cardiac death (SCD)/aborted SCD, heart failure-related death and stroke-related death (21). SCD/aborted SCD was a composite endpoint, which comprised of SCD, successful resuscitation after cardiac arrest as well as appropriate ICD work for ventricular fibrillation (VF) or ventricular tachycardia with haemodynamic instability (22). Chronic heart failure was diagnosed according to the symptoms such as shortness of breath at rest or during exertion and physical signs of fluid retention such as ankle swelling according to the New York Heart Association (NYHA) functional classification (21). The results of survival outcome of patients after ASA were obtained by the actual medical records.

### Statistical analysis

Continuous data were expressed as mean ± SD and categorical data were describe as median (interquartile range), or n (%), respectively. The different characteristics between groups were compared using the methods of unpaired Student *t*-tests, chi-squared tests, or Fisher exact test when appropriate. We stratified study participants according to their myocardial fibrosis burden into patients with myocardial fibrosis above (LGE ≥ 7.7% of LV mass) and below (LGE < 7.7% of LV mass) the median of the entire cohort. Cumulative survival curves were performed with the Kaplan–Meier method. Multivariate Cox proportional hazard regression models were used to identify whether there was an association between LGE and the long-term outcomes, and the models were corrected for age, sex, and body surface area and other clinical parameters such as the LVOT gradient before ASA, LV mass, preoperative LGE. We used the receiver operating characteristic (ROC) curve to evaluate the accuracy of LGE for the prediction of adverse outcomes. The Youden index was generated to determine the optimal cut-off value of LGE. All statistical tests were 2-tailed, and *p*-values were statistically significant at <0.05 using the SPSS statistical software, version 22.0 (SPSS Inc., Chicago, IL, United States).

## Results

### Baseline clinical and cardiac magnetic resonance characteristics

For this study, a total of 183 patients with HOCM underwent ASA with the CMR evaluation pre-ablation. The clinical and CMR characteristics at baseline were summarized in Table 1. The mean age was 51 ± 8 years, and 40.4% of patients were female. The mean LGE before ASA for the entire study population was 13.7 ± 9.5 g. Compared with the patients with lower LGE group, subjects in the higher LGE group showed more severe of NYHA class (85.7 vs. 72.8%, *p* = 0.014) and mitral regurgitation (MR, 53.9 vs. 33.0%, *p* = 0.022). There were no significant differences between groups regarding the history of hypertension, diabetes, and atrial fibrillation.

**TABLE 1:** Comparison of baseline clinical characteristics in patients with myocardial fibrosis classified by the median LGE (% of LV mass).

	Total	LGE% < 7.7 (*n* = 92)	%LGE ≥ 7.7 (*n* = 91)	*P-value*
Age, years	51 ± 8	51 ± 9	51 ± 8	0.961
Female sex	74 (40.4)	30 (32.6)	44 (48.4)	0.043
Hypertension	59 (32.2)	26 (28.3)	33 (36.3)	0.317
Diabetes	26 (14.2)	15 (16.3)	11 (12.1)	0.545
Atrial fibrillation	14 (7.7)	4 (4.3)	10 (11.0)	0.158
Chest pain	69 (37.7)	37 (40.2)	32 (35.2)	0.581
Dyspnea	160 (87.4)	76 (82.6)	84 (92.3)	0.079
NSVT	10 (7.0)	5 (6.6)	5 (7.5)	1.0
Syncope	58 (31.7)	34 (37.0)	24 (26.4)	0.168
Family history of HCM	40 (21.9)	18 (19.6)	22 (24.2)	0.565
NYHA III/IV	145 (79.2)	67 (72.8)	78 (85.7)	0.014
Moderate to severe MR	79 (43.4)	30 (33.0)	49 (53.9)	0.022
**Oral medication**				
β-Blocker	142 (77.6)	73 (79.3)	69 (75.8)	0.693
CCB	54 (29.5)	29 (31.5)	25 (27.5)	0.661
ACE inhibitor/ARB	31 (16.9)	13 (14.1)	18 (19.8)	0.411
LVOT gradient (mmHg)	95.2 ± 19.0	88.9 ± 18.2	101.4 ± 17.8	<0.001
Postablation gradient (mmHg)	26.7 ± 16.4	18.4 ± 11.8	35.2 ± 16.2	<0.001
**Baseline CMR variables**				
LAD (mm)	40.8 ± 7.3	39.7 ± 7.3	41.9 ± 7.3	0.034
Septal thickness (mm)	23.2 ± 4.8	22.4 ± 4.5	23.9 ± 5.0	0.035
LV mass (g)	176.3 ± 48.8	156.9 ± 43.7	195.8 ± 46.1	<0.001
LV mass index (g/m^2^)	100.0 ± 25.7	87.8 ± 21.0	112.4 ± 24.2	<0.001
LVEDD (mm)	45.6 ± 4.3	45.8 ± 3.9	45.5 ± 4.7	0.554
LVEDV (ml)	116.6 ± 22.5	119.0 ± 23.6	114.1 ± 21.2	0.144
LVESV (ml)	34.1 ± 11.8	31.9 ± 10.8	36.2 ± 12.4	0.014
LV EF,%	70.9 ± 7.9	73.3 ± 6.6	68.5 ± 8.4	<0.001
LGE mass (g)	13.7 ± 9.5	6.4 ± 6.1	21.1 ± 5.9	<0.001
LGE extent (% of LV mass)	7.3 ± 4.5	3.7 ± 3.1	10.8 ± 2.4	<0.001

Data are presented as the mean ± SD, number (percentage). LV, left ventricular; ACE, angiotensin-converting enzyme inhibitor; ARB, angiotensin II receptor blocker; CMR, cardiac magnetic resonance imaging; NYHA, New York Heart Association; LGE, late gadolinium enhancement; EF, ejection fraction; LVOT, left ventricular outflow tract; NSVT, non-sustained ventricular tachycardia; LVEDV, left ventricular end-diastolic volume; LVESV, left ventricular end-systolic volume; LVEDD, left ventricular end-diastolic diameter; LAD, left atrial diameter; MR, mitral regurgitation.

### Assessment of parameters by cardiac magnetic resonance

**FIGURE 1:**
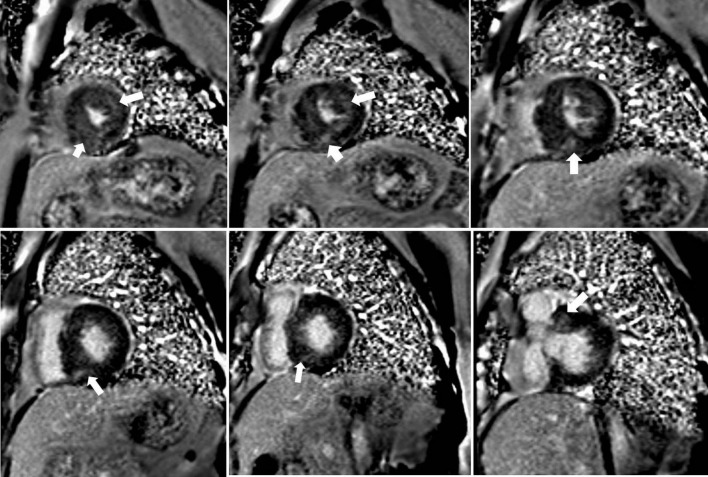
Representative short-axis late gadolinium enhancement (LGE) images in a 35-year-old male with obstructive hypertrophic cardiomyopathy showing diffuse hyperenhancement (white arrows) in the ventricular septum with 11.3% LGE compared to the LV mass.

At the pre-ablation CMR, the higher LGE group showed higher left atrial diameter (41.9 ± 7.3 vs. 39.7 ± 7.3 mm, *p* = 0.034), septal thickness (23.9 ± 5.0 vs. 22.4 ± 4.5 mm, *p* = 0.035), and LVESV (36.2 ± 12.4 vs. 31.9 ± 10.8 ml, *p* = 0.014). The LV mass (195.8 ± 46.1 vs. 156.9 ± 43.7 g) and LV mass index (112.4 ± 24.2 vs. 87.8 ± 21.0 g/m^2^) were significantly greater in the higher LGE group than the lower group (both *p* < 0.001). LGE was present in 80.9% of HCM patients (Figure 1). The higher LGE group had higher LGE mass (21.1 ± 5.9 g vs. 6.4 ± 6.1 g, *p* < 0.001) and the extent of LGE (10.8 ± 2.4% vs. 3.7 ± 3.1%, *p* < 0.001).

### Alcohol septal ablation procedural characteristics

The volumes of the alcohol during ASA were similar between the groups (2.1 ± 0.5 vs. 1.9 ± 0.6 ml, *p* = 0.171). After ASA, 32 (17.7) patients needed the temporary pacemaker implantation due to complete heart block. There was no significant difference regarding the complete heart block between the two groups (16.7 vs. 18.7%, *p* = 0.873). The LVOT gradient pre-ASA (101.4 ± 17.8 vs. 88.9 ± 18.2 mm Hg, *p* < 0.001) and post-ablation gradient (35.2 ± 16.2 vs. 18.4 ± 11.8 mmHg, *p* < 0.001) were both higher in the higher LGE group compared with the lower group. Compared to the higher LGE group, the LVOT gradient experienced the larger decrease in the lower LGE group (70.5 ± 12.9 vs. 66.3 ± 12.2 mmHg, *p* = 0.024). The procedural mortality was 0%. In the follow-up, only three patients needed permanent pacemaker implantation because of complete heart block.

### Long-term outcome at follow-up

The median time of follow-up was 6 years (range 2–11 years). Fourteen (7.7%) patients died of cardiovascular-related diseases. Eleven patients (6%) died of SCD. Three patients (1.7%) died of stroke. The comparison of patients who experienced cardiovascular-related death with the rest of the cohort is provided in Table 2. Compared with the survival patients, the patients who died were older and have more severe mitral regurgitation. The patients who died showed higher LVOT gradient (114.2 ± 10.8 vs. 93.6 ± 18.7 mmHg, *p* < 0.001) and post-ablation gradient (42.4 ± 10.3 vs. 25.5 ± 16.2 mmHg, *P* < 0.001). Furthermore, the septal thickness (27.3 ± 8.2 vs. 22.8 ± 4.3 mm, *p* = 0.001) and LV mass index (137.1 ± 23.7 vs. 96.9 ± 23.5 g/m2, *p* < 0.001) were higher in the group who died than the survival patient. The group who died showed higher LGE mass (25.5 ± 5.3 vs. 12.7 ± 9.1 g, *p* < 0.001) and more extent of LGE (11.2 ± 2.9 vs. 6.9 ± 4.5%, *p* < 0.001).

**TABLE 2:** Comparison of clinical and CMR characteristics of patients with cardiovascular-related mortality with the remainder of the cohort after ASA.

	CVD (*n* = 14)	Rest of HCM population (*n* = 169)	*P-value*
Age, years	58 ± 4	50 ± 8	0.001
Female sex	10 (71.4)	64 (37.9)	0.03
Hypertension	4 (28.6)	55 (32.5)	0.994
Diabetes	3 (21.4)	23 (13.6)	0.684
Atrial fibrillation	0 (0.0)	14 (8.3)	0.550
Chest pain	4 (28.6)	65 (38.5)	0.655
Dyspnea	12 (85.7)	148 (87.6)	1.00
NSVT	8 (57.1)	0 (0.0)	<0.001
Syncope	3 (21.4)	55 (32.5)	0.575
Family history of HCM	3 (21.4)	37 (21.9)	1.00
NYHA III/IV	11 (78.5)	134 (79.3)	0.646
Moderate to severe MR	11 (78.5)	68 (40.5)	0.013
**Oral medication**			
β-Blocker	10 (71.4)	132 (78.1)	0.808
CCB	5 (35.7)	49 (29.0)	0.822
ACE inhibitor/ARB	2 (14.3)	29 (17.2)	0.783
LVOT gradient (mmHg)	114.2 ± 10.8	93.6 ± 18.7	<0.001
Postablation gradient (mmHg)	42.4 ± 10.3	25.5 ± 16.2	<0.001
**CMR variables**			
LAD (mm)	44.2 ± 4.9	40.6 ± 7.4	0.073
Septal thickness (mm)	27.3 ± 8.2	22.8 ± 4.3	0.001
LV mass (g)	235.7 ± 46.1	171.3 ± 45.8	<0.001
LV mass index (g/m^2^)	137.1 ± 23.7	96.9 ± 23.5	<0.001
LVEDD (mm)	43.1 ± 4.1	45.9 ± 4.3	0.020
LVEDV (ml)	116.9 ± 14.2	116.6 ± 23.1	0.964
LVESV (ml)	39.2 ± 9.4	33.6 ± 11.8	0.089
LV EF,%	66.4 ± 7.5	71.3 ± 7.8	0.025
LGE mass (g)	25.5 ± 5.3	12.7 ± 9.1	<0.001
LGE extent (% of LV mass)	11.2 ± 2.9	6.9 ± 4.5	<0.001

Data are presented as the mean ± SD, number (percentage). CVD, cardiovascular death; LV, left ventricular; ACE, angiotensin converting enzyme inhibitor; ARB, angiotensin II receptor blocker; CMR, cardiac magnetic resonance imaging; NYHA, New York Heart Association; LGE, late gadolinium enhancement; EF, ejection fraction; LVOT, left ventricular outflow tract; NSVT, non-sustained ventricular tachycardia; LVEDV, left ventricular end-diastolic volume; LVESV, left ventricular end-systolic volume; LVEDD, left ventricular end-diastolic diameter; LAD, left atrial diameter; MR, mitral regurgitation.

**FIGURE 2:**
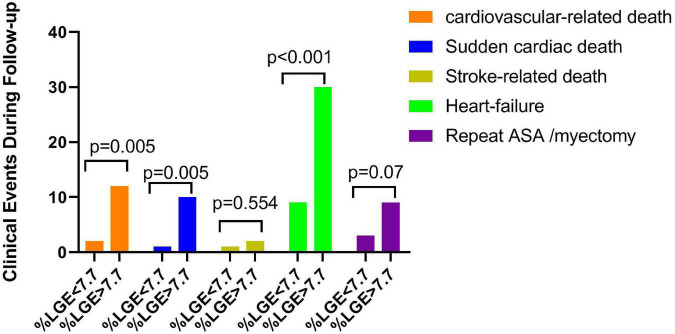
Postoperative adverse clinical events, including cardiovascular death, sudden cardiac death, stroke-related death, heart failure, and repeat ASA/myectomy, were categorized by the median late gadolinium enhancement (LGE) (% of LV mass).

**FIGURE 3:**
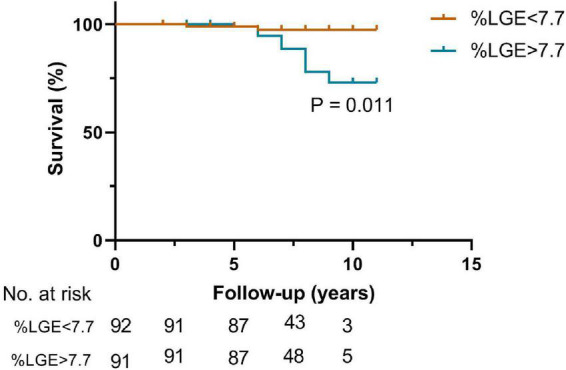
Kaplan–Meier curves describing freedom from cardiovascular mortality events in patients undergoing alcohol septal ablation with an extent of LGE < 7.7% and ≥7.7% of LV mass.

**FIGURE 4:**
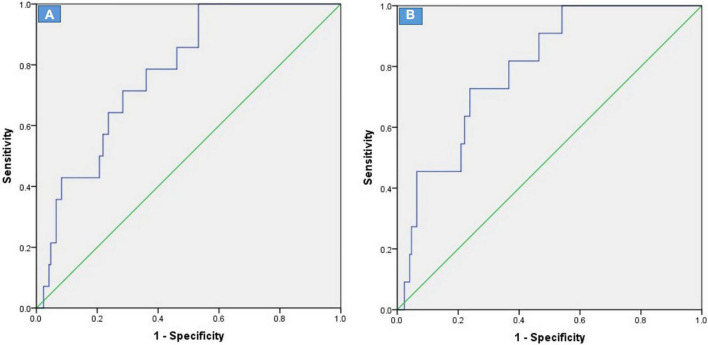
ROC curves of %LGE for estimated 6-year cardiovascular mortality **(A)** and SCD events **(B)** after alcohol septal ablation in patients with obstructive HCM.

As shown in Figure 2, compared with the lower LGE group, the higher LGE group showed more worse clinical events including the SCD (5.5 vs. 0.5%, *p* = 0.005), heart failure (33.0 vs. 9.8%, *p* < 0.001) and repeat ASA/septal myectomy (4.9 vs. 1.6%, *p* = 0.07). Kaplan–Meier survival analysis showed a significant increase in cardiovascular events (log rank = 6.399, *p* = 0.011, Figure 3). All univariate significant parameters as well as LVM and LGE were inserted into a Cox Proportional Hazards Model. Thereby, multivariate-adjusted Cox regression analyses revealed that age [HR 1.142 (1.059–1.230), *p* = 0.001] and LGE [HR 1.170 (1.074–1.275), *p* < 0.001] were independent predictors of cardiovascular mortality during follow-up (Table 3). In addition, age [HR 1.129 (1.039–1.226), *p* = 0.004] and LGE [HR 1.189 (1.073–1.317), *p* = 0.001] were also independent predictors of SCD events in HCM patients undergoing ASA during follow-up (Table 4) (As shown in Figure 4, the ROC analysis indicated that LGE had reasonable accuracy for prediction of adverse outcomes as well as SCD events). The cut-off values of LGE of estimated 6-year cardiovascular mortality and SCD were 7% of LV mass (sensitivity 100% and specificity 46.8%, ROC area 0.774, 95% CI 0.707–0.833, *p* < 0.001) and 10% of LV mass (sensitivity 72.73% and specificity 76.16%, ROC area 0.793, 95% CI 0.727–0.849, *p* < 0.001) respectively.

**TABLE 3:** Univariate and multivariate predictor models for the composite endpoint of cardiovascular-related death in HCM patients undergoing ASA.

	Univariate model			Multivariate model		
	HR	95% CI	*P-value*	HR	95% CI	*P-value*
Age	1.129	1.054–1.210	0.001	1.142	1.059–1.230	0.001
Gender	3.429	1.076–10.936	0.037			
Septal thickness	1.090	1.025–1.158	0.006			
LAD	1.088	1.008–1.175	0.032			
LVEDD	0.863	0.770–0.968	0.012			
LVOT gradient	1.038	1.013–1.064	0.003			
Postablation gradient	1.075	1.039–1.112	<0.001			
LVM	1.019	1.009–1.028	<0.001			
LGE mass	1.148	1.073–1.228	<0.001	1.170	1.074–1.275	<0.001

LAD, left atrial diameter; LVEDD, left ventricular end-diastolic diameter; LVOT, left ventricular outflow tract; LVM, left ventricular mass; LGE, late gadolinium enhancement; ASA, alcohol septal ablation.

**TABLE 4:** Univariate and multivariate predictor models for SCD events in HCM patients undergoing ASA.

	Univariate model			Multivariate model		
	HR	95% CI	*P-value*	HR	95% CI	*P-value*
Age	1.107	1.021–1.201	0.014	1.129	1.039–1.226	0.004
Gender	4.283	1.097–16.721	0.036			
Septal thickness	1.142	1.036–1.259	0.008			
LAD	1.038	0.956–1.127	0.378			
LVEDD	0.834	0.731–0.951	0.007			
LVOT gradient	1.063	1.021–1.106	0.003			
Postablation gradient	1.054	1.015–1.1095	0.006			
LVM	1.022	1.010–1.035	<0.001			
LGE mass	1.162	1.074–1.256	<0.001	1.189	1.073–1.317	0.001

LAD, left atrial diameter; LVEDD, left ventricular end-diastolic diameter; LVOT, left ventricular outflow tract; LVM, left ventricular mass; LGE, late gadolinium enhancement; ASA, alcohol septal ablation.

## Discussion

In this study, we assessed the impact of myocardial fibrosis measured by CMR on ASA outcomes in patients with HOCM. Our results suggested that those who presented with higher amount of LGE had greater incidence of long-term cardiovascular events compared with those with lower LGE group. Second, after multivariate adjustment, the degree of LGE was an independent risk factor predicting the long-term outcomes, confirming that the LGE is an available important marker of prognosis after ASA, thus more scientific follow-up plans and monitoring during out-hospital could be made to prevent from cardiac complications after ASA for those with higher amounts of LGE.

Late gadolinium enhancement has been related to adverse clinical outcomes in the progression of hypertrophic cardiomyopathy (19). We extend here those findings to patients with HCM undergoing ASA treatment. Our study is the first study to date to evaluate the prognostic value of LGE-CMR on perioperative and long-term survival after ASA for obstructive HCM. We demonstrated that preoperative LGE was the independent predictor of cardiovascular mortality after ASA on multivariate analyses. Considering that the majority of cardiovascular deaths in our study were sudden death, the presence of LGE increased the risk of sudden cardiac death in patients with HOCM after ASA, which was consistent with Rigopoulos et al. result (12). The mechanism of arrhythmias in HCM after ASA remains unclear. The areas of LGE in HCM had been proved to exhibit both depolarization and repolarization abnormalities which may trigger malignant ventricular arrhythmia. More extensive myocardial fibrosis was associated with a higher burden and prolonged bursts of ventricular tachyarrhythmia, which may cause the occurrence of SCD (15, 23, 24).

Previous studies have demonstrated there was a very low incidence of ventricular arrhythmias and sudden cardiac death after alcohol septal ablation (12, 25). In our study, sudden death occurred in 11 (6%) patients with an annual mortality rate of 0.89 per 100 patient-years and cardiovascular death occurred in 14 patients with an annual mortality ratio of 1.13 per 100 patient-years which was similar to veselka et al. results (26) that the event rate of sudden death and cardiovascular related mortality events was 0.98 and 1.16 per 100 patient-years respectively. The lower occurrence of significant ventricular arrhythmias or SCD may be related to the improved haemodynamic conditions and the regression of LV mass in the long-term after ASA. LVOT obstruction is also regarded as a clinical risk factor for sudden cardiac death (27). Ommen et al. demonstrated that the reduction of LVOT gradient contributed to the better survival from sudden cardiac death compared with patients with HOCM who only received medical drugs (28). The possible mechanism would include the reduction of arrhythmogenic substrate production and normalization of left ventricular pressure after relieving the obstruction of LVOT in patients undergoing ASA treatment.

Alcohol septal ablation has been shown to reduce LVOT gradient and alleviate symptoms safely and effectively in symptomatic patients with HOCM (29). However, whether myocardial fibrosis has an effect on the LVOT gradient reduction and long-term prognosis is still unclear. In our study, we showed that high amounts of LGE by CMR were associated with more severe HCM phenotype and LV remodeling including higher LV mass index and lower EF. Moreover, the group with higher LGE had greater baseline LVOT gradient as well as residual gradient after ASA, which was consistent with the recent findings that the LGE + group had higher residual LVOT gradient than the LGE-group (30). Jensen et al. also demonstrated that the baseline and residual LVOT gradients were both higher in HCM patients with more pronounced hypertrophy (31). Myocardial fibrosis is the process of excessive deposition of extracellular matrix proteins secondary to the hypertrophy in response to the pressure overload and may be a determinant of disease progression and clinical prognosis (16, 20, 32).

Several published studies have shown the factors associated with unfavorable outcomes undergoing ASA. Sorajja et al. showed that residual LVOT gradients after ASA were associated with reduced survival (2). Jensen et al. found that severe septal hypertrophy before ASA was the marker of poor outcome after ASA (31). Veselka et al demonstrated that early postdischarge LVOT gradient ≥30 mm Hg and baseline septum thickness were the independent predictors of cardiovascular events in the patients after ASA (33). In our study, when the LGE was not included in the multivariate model, the baseline LVOT gradient and LVM were independent predictors of cardiovascular mortality, however, after the LGE was added to the model, age and LGE before ASA were the independent predictors of mortality events. Because the amount of LGE was an important marker of increased risk for SCD and the development of heart failure (7), identification of LGE may provide additional information and proof for those patients with higher LGE who may benefit from implanted cardioverter-defibrillator (ICD) therapy after receiving ASA treatment (34).

Although the residual LVOT gradient and the severity of preoperative MR were higher in patients with the worse clinical outcome, the parameters were not predictors of outcome on multiple regression analysis, which was consistent with Chang et al. results (3). The univariate association of residual LVOT gradient and outcome may be attribute to the need for a larger infarct in certain cases with a large culprit septal area to achieve successful results. Furthermore, in accordance with Faber et al.’s results (4), the parameter of peak creatine kinase as the indicator of infarct size was not related to ASA outcome in the multivariate analysis. The reason may be explained that the larger infarct size was associated with the larger decrease of septal mass in the short term and the long term outcome may be attributed to the reduction of LVOT gradient and the concomitant decrease in LV wall stress (35). Myocardial fibrosis may lead to the increase of LV wall stress and portend susceptibility for progression to heart failure as well as risk for arrhythmic sudden death.

### Clinical implications

Currently, the clinical evaluation of patients with HCM when receiving ASA treatment is based mainly the symptoms and LVOT gradient. Because myocardial fibrosis progresses slowly with the LV mass in HCM, which is also correlated with the poor clinical outcomes such as heart failure and the future implantation of ICD (19), our findings proved that myocardial fibrosis also predicts mortality in patients after ASA, suggesting the LGE–CMR could potentially be useful for selecting the operative approach for reducing the risk of mortality. Patients with >7% amount of LGE, indicating lower survival and higher risk of heart failure and residual LVOT gradient could be carefully monitored to prevent from cardiovascular events. Moreover, the higher risk of sudden cardiac death in HCM patients with higher extent of LGE indicates that they could potentially benefit from ICD device to improve long-term survival (22).

## Limitation

Firstly, this was a retrospective, single-center study with a modest number of events. There is potential for over-fitting in statistical analysis. Furthermore, because the age range of the patients was about 43–59 years old in our study, whether the LGE affects the prognosis of patients with HCM after ASA in other age groups (such as adolescents and elderly) will be tested in the next study. Secondly, we did not enroll the patients who received ICD treatment before ASA because of the contraindication of CMR examination. Moreover, there were lower incidence of ICD implantation for the patients with higher risk of SCD after ASA because of misgivings about potential complications and cost in Fuwai Hospital, which was consistent with other results in our cohort (36) as well as other cohort in China (29). The potential for selection bias may exist in our study. However, the results obtained from this kind of HCM population may reflect the natural disease course to a certain extent. Thirdly, genetic data were not routinely performed. The relationship of genetic data to the clinical outcome in patients after ASA may be studied in the future study. Fourthly, at present, there is no general consensus on the protocols of LGE examination in HCM. The protocol of the LGE examination by CMR commonly used in Fuwai Hospital in this study may be different with other institutions. The possibility that different protocols introduce bias in LGE quantification cannot be ruled out. Finally, our study focused only the survival data, whereas did not perform to follow-up the patients’ functional status and CMR-related improvement, which may be considered in the future study.

## Conclusion

Our study demonstrates that the preoperative LGE by CMR can predict the long-term cardiovascular mortality in patients undergoing ASA. Therefore, the evaluation of LGE pre-ablation could act as a new marker for risk stratification in patients with HCM undergoing ASA.

## Data availability statement

The original contributions presented in this study are included in the article/supplementary material, further inquiries can be directed to the corresponding authors.

## Ethics statement

The studies involving human participants were reviewed and approved by the Institutional Review Committee of Fuwai Hospital, China. The patients/participants provided their written informed consent to participate in this study.

## Author contributions

YC and SQ conceived and designed the manuscript. YC analyzed the data and wrote the manuscript. JY, YZ, XZ, and WL edited the manuscript. All authors contributed to the article and approved the submitted version.
